# *Centella asiatica* Extract Improves Behavioral Deficits in a Mouse Model of Alzheimer's Disease: Investigation of a Possible Mechanism of Action

**DOI:** 10.1155/2012/381974

**Published:** 2012-02-15

**Authors:** Amala Soumyanath, Yong-Ping Zhong, Edward Henson, Teri Wadsworth, James Bishop, Bruce G. Gold, Joseph F. Quinn

**Affiliations:** ^1^Department of Neurology, Oregon Health & Science University, Portland, OR 97239, USA; ^2^Department of Neurology, Portland VA Medical Center, Portland, OR 97239, USA

## Abstract

*Centella asiatica* (CA), commonly named gotu kola, is an Ayurvedic herb used to enhance memory and nerve function. To investigate the potential use of CA in Alzheimer's disease (AD), we examined the effects of a water extract of CA (GKW) in the Tg2576 mouse, a murine model of AD with high *β*-amyloid burden. Orally administered GKW attenuated *β*-amyloid-associated behavioral abnormalities in these mice. *In vitro*, GKW protected SH-SY5Y cells and MC65 human neuroblastoma cells from toxicity induced by exogenously added and endogenously generated *β*-amyloid, respectively. GKW prevented intracellular *β*-amyloid aggregate formation in MC65 cells. GKW did not show anticholinesterase activity or protect neurons from oxidative damage and glutamate toxicity, mechanisms of current AD therapies. GKW is rich in phenolic compounds and does not contain asiatic acid, a known CA neuroprotective triterpene. CA thus offers a unique therapeutic mechanism and novel active compounds of potential relevance to the treatment of AD.

## 1. Introduction

*Centella asiatica *(L.) Urban, family Apiaceae (CA), is known as *Mandookaparni* or *Brahmi* in Ayurvedic medicine. It is highly regarded as a “rasayana” or rejuvenating herb [1] and is reputed to increase intelligence and memory [1]. The dried herb has enjoyed growing popularity in the USA and other Western countries, where it is sold as the dietary supplement “gotu kola” [2].

 Cognitive effects of the aqueous extract of CA (100–300 mg/kg/day) have been evaluated in several rodent studies using standard tests including shuttle box, step-through paradigm, elevated plus maze, and passive avoidance tests. CA extract markedly improved learning and memory of wild-type rats [3], rats subjected to CNS toxicity (intracerebroventricular streptozotocin) [4], and pentylenetetrazole (PTZ) kindled rats [5]. When administered to neonatal mice from day 15 to 30 postpartum, the extract caused significant enhancement in learning efficiency and spatial memory with no effects on locomotor function [6]. Direct neurotropic effects of CA have also been reported. CA aqueous extract caused significant increases in dendritic arborization of apical and basal dendrites in hippocampal neurons of neonatal mice [6] and both adult [7] and neonatal [8] rats. These studies, performed in diverse settings, show that CA water extract has biological effects of relevance to memory, learning, and aging, and potentially to disease progression in Alzheimer's disease (AD).

 The present study examines the effect of a water extract of CA on behavioral deficits in the Tg2576 transgenic mouse, a murine model of AD. The Tg2576 or “Hsiao” transgenic mouse has been described in detail [9, 10] and is one of the most widely used animal models of AD. A mutant human amyloid precursor protein (APP) gene inserted into the genome gives rise to age-dependent hippocampal and cortical *β*-amyloid (A*β*) plaques similar to AD pathology. Plaques are not histologically evident until 10–12 months of age, and plaque pathology is confined to the hippocampus and cerebral cortex. In other words, the age and region dependence of pathology in AD are nicely recapitulated in this strain. Other features of AD reproduced in this strain are astrocytic and microglial activation surrounding the A*β* plaques and dystrophic changes in neurites in the vicinity of plaques [11]. Importantly, these mice display abnormalities in standard behavioral tests. Open-field behavior has been shown to distinguish Tg2576 from wild-type mice, with Tg2576 mice more active in the open field than their wild-type littermates [12]. Hippocampal dysfunction, resulting in impaired spatial memory, is evident in the Morris water maze and has been repeatedly shown to distinguish aged, A*β* plaque-bearing Tg2576 from wild-type mice [9, 13]. These behavioral tests were utilized in the present study.

 In addition to *in vivo* studies, the present work examined possible mechanisms underlying CA effects in the Tg2576 mouse, using *in vitro* models. The mechanisms examined include cholinesterase inhibition and neuroprotectant effects against oxidative damage, glutamate toxicity, and beta amyloid toxicity. The ingredient compounds in CA aqueous extract were also investigated.

## 2. Materials and Methods

### 2.1. Aqueous Extraction of CA

Dried CA was purchased from Oregon's Wild Harvest, Sandy, OR (Batch no. GOT-10072C-OGA). The identity of the herb was verified by means of visual examination and by comparing its thin layer chromatographic profile with that reported in the literature [14]. A dried water extract (GKW) was prepared by refluxing CA (120 g) with water (1.5 L) for 2 hr, filtering to remove plant debris and freeze-drying to yield a residue (11.5 g).

### 2.2. Chemical Analysis of CA Extracts

Water and ethanolic extracts of CA were compared by high-performance liquid chromatography coupled to UV detection (LC-UV) and mass spectrometry (LC-MS). The extracts were chromatographed alongside commercial reference standards of asiatic acid, madecassic acid, asiaticoside, and madecassoside (ChromaDex, Irvine, CA). Analysis was performed using LC-MS in negative ion mode on an LCQ Advantage ion trap mass spectrometer (Thermo Electron, San Jose, CA) with an in-line Surveyor autosampler and HPLC (Thermo Electron) coupled to a Surveyor Photodiode array detector (Thermo Electron). HPLC used an Aquasil 5 *μ*m C18 150 × 2.1 mm column eluting with a gradient of acetonitrile in water both with 0.01% formic acid (acetonitrile 5% to 25% in 20 min, to 40% at 35 min, 60% at 40 min, 75% at 45 min and then returning to starting conditions).

### 2.3. Administration of GKW to Mice

Fifteen Tg2576 and 20 wild-type 20-month-old female mice were committed to this experiment. Approximately half of each genotype group was administered GKW in the drinking water at a dose of 2 mg/mL of water, with water bottles changed every other day, calculated to yield 200 mg/kg/day, a dose previously shown to improve memory in wild-type rats [3]. The GKW-treated group included 8 Tg2576 and 10 wild-type mice; the untreated group included 7 Tg2576 and 10 wild-type mice. Treatment was continued for 2 weeks, the duration of treatment in published reports showing behavioral effects in wild-type animals [3]. Open-field behavior and Morris water maze testing were performed at the end of this period.

### 2.4. Open-Field Testing of Mice

Mice were placed in the center of a square arena (38 × 38 × 64 cm high) constructed of white acrylonitrile butadiene styrene for two 5-minute open-field sessions on each of three consecutive days. Distance moved and velocity of each mouse were continuously tracked with a digital camera and “ANY-maze” software (ANY-Maze, Inc., Greensburg, PA). After a 5-minute epoch was recorded, the mouse was returned to the cage for 5 minutes and then retested. For each mouse, two tests were conducted on each of 3 days.

### 2.5. Morris Water Maze Testing of Mice

A subset of the treated animals (5 untreated wild-type, 5 GKW-treated Tg2576, and 5 untreated Tg2576) was also tested in the Morris water maze, a well established assay of hippocampal spatial memory [15]. This paradigm tests the animal ability to learn and remember the spatial location of a platform submerged 1 cm in a 109 cm circular pool of opaque water. The mice were habituated to the examination room and holding cages for two days and then received two days of “nonspatial training” before commencement of hidden platform testing. Each nonspatial training trial comprised placing the mouse on the submerged platform for 60 sec and then placing the mouse in close proximity (within 2-3 cm) to the platform and allowing it to climb onto the platform from the water. Training on the hidden platform water maze task began 24 hr after the last habituation trial. At this stage, curtains were opened to permit the mice visual access to extra maze cues in the room surrounding the maze. Hidden platform training was conducted over 16 trials on 4 consecutive days (4 trials/day). The platform remains in a fixed position throughout hidden platform training. During a given trial, the mouse is introduced into the pool at one of four randomly chosen start points (N, S, W, and E) and allowed 60 sec to find the platform. All trials were monitored by a video camera positioned above the pool and the behavior of each mouse was acquired by a computerized video tracking system (ANY-Maze software). Dependent measures acquired in each trial include the escape latency (i.e., time to find the platform, in sec), the cumulative distance (in cm) of the mouse from the platform, and swim speed (in cm/sec). No mice in this group needed to be excluded based on difficulty in swimming, in climbing onto the platform, or exhibiting abnormal swimming patterns or persistent floating.

### 2.6. Brain Levels of Soluble and Insoluble A*β*

Brain levels of A*β* were measured in experimental mice at the conclusion of behavioral testing described above. Tg2576 mice were sacrificed and brains rapidly harvested, divided, and frozen until analysis. Cortical tissue was then homogenized in buffered saline and protease inhibitors and ultracentrifuged to yield a “soluble” fraction, as we have reported previously [16–18]. The remaining pellet was rehomogenized and incubated in guanidine and buffer and subsequently ultracentrifuged to yield a “fibrillar” fraction. Concentrations of A*β*_1-40_ and A*β*_1-42_ were determined in each supernatant using commercial ELISA kits, which distinguish the two isoforms (BioSource international, Camarillo, CA). Total protein in each fraction was determined by the Bradford method.

### 2.7. Neurotoxicity Induced by Extracellular A*β*

SHSY5Y neuroblastoma cells were grown in DMEM/F12 medium (from Gibco) containing fetal calf serum (FCS; 10%), streptomycin sulphate (100 *μ*g/mL), and penicillin G (1000 U/mL) in a humidified air/5% CO_2_ chamber at 37°C. On day 1, the cells were plated in 24-well plates (100,000 cells/well).

On day 4, cells were washed with FCS-free medium and further incubated in FCS-free DMEM/F12 containing neuroblastoma growth supplement N-2 (1%; Gibco). GKW (0–200 *μ*g/mL) was added and incubated overnight. On day 5, cells were exposed to A*β*_25-35_ (American Peptide Company) at 20 or 50 *μ*M for 48 hrs in the presence of GKW. For this assay, fibrillar A*β* solution was freshly prepared by sonicating a solution of A*β*_25-35_ in medium for 1 minute prior to addition to cell cultures. On day 7, the supernatant was harvested for LDH cell integrity assay; LDH release is a marker of cell damage [19]. Fresh medium was added and cell number assessed using CellTiter-Blue reagent (resazurin, Promega), incubating for 2 hours and reading fluorescence at 560 nm excitation and 590 nm emission.

### 2.8. Intracellular A*β*-Induced Neurotoxicity in MC65 Cells

MC65 cells are an established neuroblastoma cell line that conditionally express the C-terminal fragment of amyloid precursor protein (APP CTF) [20]. Following withdrawal of tetracycline from the media, the cells generate endogenous A*β* and die within 3 days. Expression of APP CTF in MC65 cells leads to the formation of intracellular A*β* aggregates. Previous studies have shown a strong correlation between these aggregates and subsequent cytotoxicity that is associated with oxidative stress [21]. MC65 cells were cultured and maintained in MEME*α* supplemented with 10% FBS (Gibco-BRL, Carlsbad, CA) and 1 *μ*g/mL tetracycline (Sigma-Aldrich, St. Louis, MO) as previously described [21, 22]. Confluent cells were trypsinized, washed with PBS, resuspended in OptiMEM without phenol red (Gibco/BRL, Carlsbad, CA), and plated at 25,000 cells/well in 48-well plates in fresh medium containing vehicle or desired concentrations of GKW, in the absence of tetracycline. Cell viability was measured at 2.75 days using the CellTiter 96 Aq_ueous_ Non-Radioactive Cell Proliferation Assay (Promega Corporation, Madison, WI) according to manufacturer's instructions. Experiments were carried out in triplicate wells for each condition and repeated 1-2 times.

### 2.9. APP CTF Aggregation in MC65 Cells

Cells were plated in culture dishes without tetracycline in the presence or absence of 100 *μ*g/mL GKW. Cells were harvested at 2 days and lysates prepared by sonication and heating in Laemmli sample buffer. Samples were reduced then separated on tricine gels, transferred, and western blotted using mouse monoclonal antibody 6E10 that recognizes A*β*_1-17_.

### 2.10. A*β*-Induced Nitric Oxide in Macrophages

RAW 264.7 cells were cultured in complete phenol red-free DMEM containing 50 units/mL of penicillin, 50 mg/mL of streptomycin, 44 mM sodium bicarbonate, and 10% fetal bovine serum (complete medium) at 37°C in humidified air containing 5% CO_2_. Cells were plated in 24-well plates (approximately 1 × 10^5^ cells/well) and cultured for 2 days until cells reached 80% confluency. Cells were washed, treated with fresh complete media containing various concentrations of GKW for 1 hour, and then induced with LPS (100 ng/mL) or A*β*_1-42_ (20 *μ*M) for an additional 24 hours. A*β* fibril formation was induced by incubating A*β* at 37°C overnight prior to addition to cell cultures. Nitrite was determined in supernatants from treated macrophages using Griess reagent as described previously [23, 24]. Nitrite concentrations were determined using dilutions of sodium nitrite in complete medium as a standard.

### 2.11. Anticholinesterase Activity

Cholinesterase activity was measured by the method of Ellman et al. [25], which is based on the formation of a yellow reaction product when thiocholine is liberated from acetylthiocholine and combines with the test reagent, dithiobisnitrobenzoic acid (DTNB). Potential cholinesterase inhibitory effect of GKW was tested directly in an assay using mouse plasma cholinesterase. DTNB buffer (80 *μ*L) was added to a 96-well plate, followed by mouse plasma (5 *μ*L), and then test agent (5 *μ*L), consisting of GKW solution (2.5, 25, and 250 *μ*g/mL), neostigmine (2.5 *μ*g/mL; positive control), or buffer (negative control) and the solution was warmed to 37°C. The substrate, acetylthiocholine, was then added and absorbance recorded at 450 nm for 5 minutes.

### 2.12. Glutamate-Induced Neurotoxicity

Potential protection against glutamate-induced toxicity was examined in cortical neurons obtained from neonatal Sprague-Dawley rats. On day 1, cells were plated (200,000 cells per well) in 48-well plates using neurobasal medium supplemented with B27 (2%) containing antioxidants, 2 mM L-glutamine (Gibco). On day 4, the cells were treated with Ara-C (5 *μ*M) to remove glial cells. On day 7, cells were washed with prewarmed CSF buffer (2x, 0.5 mL/well) then incubated with neurobasal medium, plus L-glutamine (2 mM) and B27 (2%) minus antioxidants, overnight. On day 8, sodium glutamate (0–1000 *μ*M) with or without GKW (0–200 *μ*g/mL) was added to the medium and the cells incubated overnight. On day 9, fresh medium was added and cell number evaluated using CellTiter Blue reagent (resazurin, Promega), incubating for 2 hours and reading fluorescence in a fluorimeter at 560 nm excitation and 590 nm emission.

### 2.13. Antioxidant Activity

Potential antioxidant effects of GKW were assessed* in vitro* in SH-SY5Y cells treated with hydrogen peroxide as an oxidative stressor. SHSY5Y neuroblastoma cells were grown in DMEM/F12 medium (from Gibco) containing fetal calf serum (FCS; 10%), streptomycin sulphate (100 *μ*g/mL), and penicillin G (1000 U/mL) in a humidified air/5% CO_2_ chamber at 37°C. On day 1, the cells were plated in 24-well plates (100,000 cells/well). On day 4, medium was replenished with fresh medium containing NGF (10 ng/mL). After 24 hours (day 5), GKW (0, 50, 100, and 200 *μ*g/mL) was added and the cells incubated overnight. On day 6, medium containing GKW was replaced with fresh medium containing less FCS (2%) and NGF (10 ng/mL). Cells were treated with a range of concentrations of H_2_O_2_ (0–500 *μ*M) for 2-3 hours. Medium containing peroxide was removed and cells incubated overnight in fresh medium containing FCS (2%) and NGF (10 ng/mL). On day 7, supernatant was harvested for lactate dehydrogenase (LDH) cell integrity assay (102). Fresh medium was added and cell number evaluated using CellTiter Blue reagent (resazurin, Promega), incubating for 2 hours and reading fluorescence at 560 nm excitation and 590 nm emission.

## 3. Results and Discussion

The water extractable compounds (GKW) represented approximately 10% of the dry weight of CA herb. There was no difference in water consumption between control animals and animals receiving water containing GKW.

**Figure 1:**
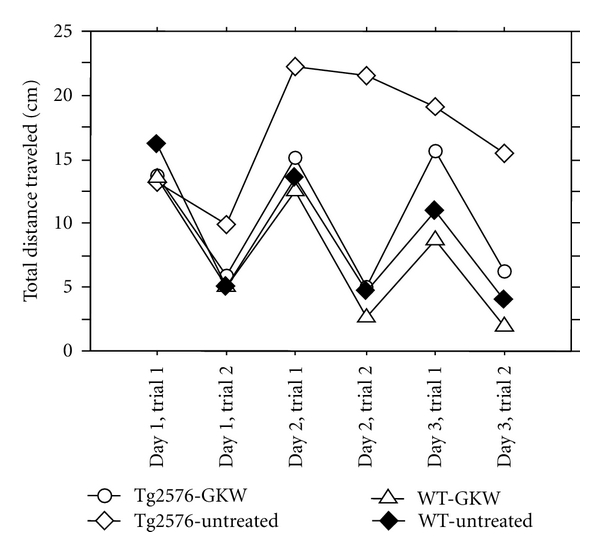
Open-field assay: effect of GKW on total distance travelled. Wild-type mice, both treated (triangle) and untreated (filled diamond), are less active on the second trial of each day, presumably due to habituation. Untreated Tg2576 mice (open diamond), in contrast, fail to habituate to the surroundings. GKW-treated Tg2576 mice (circles) explore in a manner similar to wild-type mice, with their data overlapping the two wild-type groups. From statistical analysis (ANOVA), *P* = 0.02 for difference between untreated (*n* = 7) and GKW-treated (*n* = 8) Tg2576 mice; error bars omitted for clarity.

Open-field testing (Figure 1) showed that GKW treatment caused an improvement in behavioral abnormalities seen in Tg2576 mice. Wild-type mice, both GKW treated and untreated, were less active on the second trial of each day, presumably due to habituation. Untreated Tg2576 mice, in contrast, failed to habituate to the surroundings. However, GKW-treated Tg2576 mice explored in a manner similar to wild-type mice, with their data overlapping the two wild-type groups (*P* = 0.02 for difference between untreated and GKW-treated Tg2576 mice by ANOVA). “Normalization” of open-field behavior in Tg2576 mice has also been reported with an intervention that suppresses soluble A*β* levels [12].

**Figure 2:**
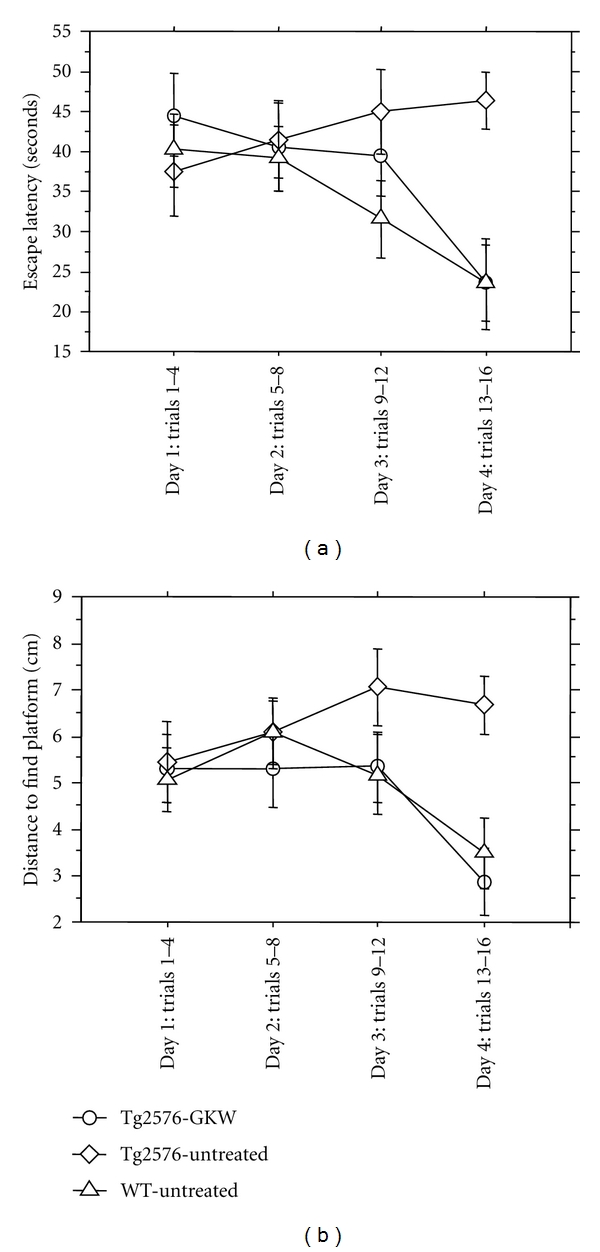
GKW effects in the Morris water maze. Mean ± SEM of (a) escape latency and (b) distance traveled to find platform is shown for each day of testing. Wild-type animals exhibit a “learning curve” requiring less time and less distance to find the hidden platform with repeated trials, while untreated Tg2576 mice require equivalent time and distance despite repeated exposures. In contrast, the GKW-treated mice learn in a manner similar to the wild-type animals, with latency and distance traveled to find the platform declining with repeated exposures. On day 4, time to find the platform was significantly greater for untreated Tg2576 mice than for wild-type (*P* = 0.002) or GKW-treated Tg2576 mice (*P* = 0.004). Distance traveled to find the platform was also significantly greater on day 4 for untreated Tg2576 mice compared to wild-type (*P* = 0.006) or GKW-treated Tg2576 mice (*P* = 0.003).

In the Morris water maze paradigm (Figure 2), GKW treatment improved the impaired learning ability evident in Tg2576 mice. Wild-type animals exhibit a “learning curve” requiring less time and less distance to find the hidden platform with repeated trials, while untreated Tg2576 mice require equivalent time and distance despite repeated exposures. In contrast, the GKW-treated Tg2576 mice learn in a manner similar to the wild-type animals, with latency and distance traveled to find the platform declining with repeated exposures. On day 4, time to find the platform was significantly greater for untreated Tg2576 mice than for wild-type (*P* = 0.002) or GKW-treated Tg2576 mice (*P* = 0.004). Distance traveled to find the platform was also significantly greater on day 4 for untreated Tg2576 mice compared to wild-type (*P* = 0.006) or GKW-treated Tg2576 mice (*P* = 0.003). There was no significant difference between groups in the “visible platform” control for sensorimotor function (data not shown).

**Figure 3:**
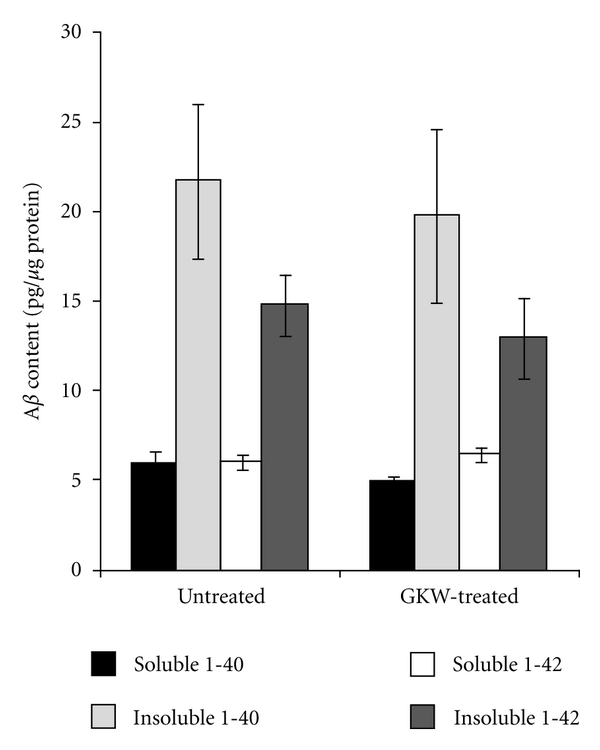
Soluble and insoluble A*β* in cortical tissue from treated and untreated mice. Mean values ± SEM are shown. Treated and untreated Tg2576 mice did not differ significantly in levels of any of the measured isoforms of A*β*.

Since treatment with GKW ameliorated a spatial memory impairment in Tg2576 mice, which is specifically associated with the appearance of A*β* plaques, without producing any change in wild-type mouse memory, it would appear that the observed effect of GKW is specific to A*β*. However, Figure 3 shows that there were no significant differences between levels of any of the forms of A*β* in treated and untreated Tg2576 mice. This is in contrast to results obtained in PSAPP mice, a model for Alzheimer's disease (AD) where mice express both amyloid precursor protein and presenilin 1 mutations, in the long term (8 months). In these mice, administration of CA extract displayed *in vitro* antioxidant effects and also reduced beta amyloid plaque burden [26]. The PSAPP mice develop amyloid plaque pathology at an earlier age than Tg2576 [27], permitting more rapid completion of antiamyloid experiments. However, loss of the age- and region-dependence of pathology diminishes the fidelity of this strain to some extent. Since GKW treatment attenuated the neurologic consequences of abnormal A*β* deposition in Tg 2576 mice without changing A*β* levels *per se*, the ability of GKW to modulate the toxic effects of A*β* were pursued *in vitro*, with an emphasis on mechanisms which are either independent of or “downstream” from A*β*. 

**Figure 4:**
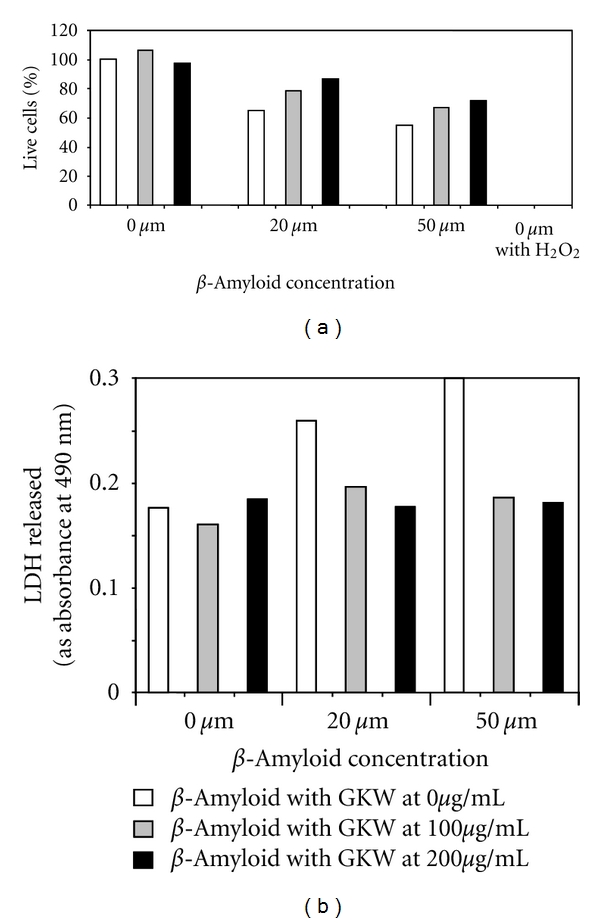
(a) GKW at 100 and 200 *μ*g/mL shows a modest protective effect on SH-SY5Y cells from A*β* toxicity (beta amyloid 25–35) *in vitro*. The % of live cells is decreased on treatment with A*β*, an effect which is attenuated by GKW. Hydrogen peroxide 500 *μ*M is used as a 0 cell viability control. (b) GKW at 100 and 200 *μ*g/mL protects SH-SY5Y cells from A*β* toxicity (beta amyloid 25–35) *in vitro*—LDH release. The amount of LDH (lactate dehydrogenase) released is increased on treatment with A*β*, an effect which is attenuated by GKW. LDH release is a marker of cell damage.

**Figure 5:**
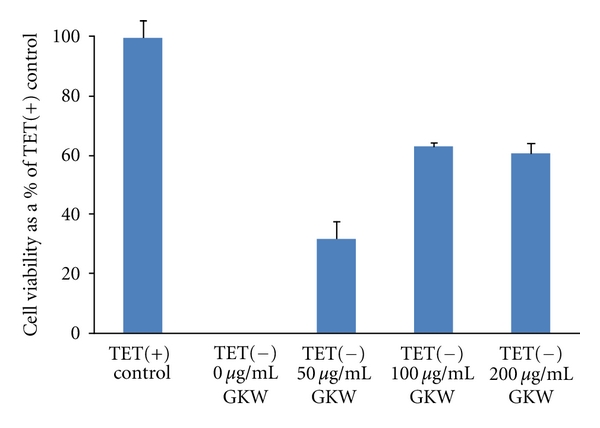
Effect of GKW on survival of MC65 cells following tetracycline withdrawal. Cell viability is expressed as a % of the cell growth obtained in control cultures containing tetracycline, TET(+). On withdrawal of tetracycline from the media, TET(−), the cells generate endogenous A*β* and die within 3 days. In the absence of GKW, cell survival in TET(−) cultures is zero. GKW dose-dependently protects MC65 cells from cell death in TET(−) cultures (**P** < 0.01 at 50, 100, and 200 *μ*g/mL compared to 0 *μ*g/mL GKW; Student's *t*-test). Mean values of cell viability ± SD are shown.

 In preliminary experiments, GKW showed a moderate protective effect against toxicity due to exogenously added A*β* in SH-SY5Y human neuroblastoma cells *in vitro* (Figure 4(a)). Lactate dehydrogenase (LDH) release from these cells, which is inversely related to cell viability, was reduced in the presence of GKW (Figure 4(b)). The effect of GKW on toxicity due to endogenously generated A*β* was investigated in MC65 human neuroblastoma cells. GKW added to the cell culture medium prevented MC65 cell death following tetracycline withdrawal, in a dose-dependent manner (Figure 5). Evidence from Western blots indicated that GKW may prevent the aggregation of A*β* in these cells. In a related study (data not shown), GKW inhibited A*β*-induced nitric oxide (NO) production in the RAW 264.7 macrophage cell line. Interestingly, GKW inhibited NO production induced by A*β* but did not influence LPS-induced NO levels in these cells. Taken together, these data indicated that components in GKW are able to modulate the toxic effects of A*β*.

Other potential mechanisms by which GKW may have improved cognitive function in the Tg2576 mice were also investigated but yielded negative results. No direct inhibitory effect of GKW (2.5 to 250 *μ*g/mL) on cholinesterase activity *in vitro* was observed, whereas robust inhibition was observed using the positive control neostigmine. Effects of GKW on glutamate toxicity to rat cortical neurons were investigated. GKW (100 or 200 *μ*g/mL) was not directly toxic to rat cortical neurons nor did it protect the cells from toxicity induced by 250 and 1000 *μ*M glutamate (cell viability 30% and 25% of control, resp.). To examine potential antioxidant effects of GKW, SHSY5Y neuronal cells were exposed to H_2_O_2_, which showed dose-dependent toxicity to SH-SY5Y cells over the range 125–500 *μ*M. GKW (50–200 *μ*g/mL), while not toxic to the cells, did not protect against toxicity at any of the peroxide concentrations tested. Thus, GKW does not appear to possess antioxidant effects.

Five drugs are currently FDA-approved for the symptomatic treatment of AD, targeting mechanisms of unclear relationship to the primary neurodegenerative process. The first four drugs (tacrine, donepezil, rivastigmine, and galantamine) are acetylcholinesterase inhibitors, which act by augmenting cholinergic neurotransmission [28]. Each of these drugs has shown improved cognitive outcomes in treated AD patients compared to placebo-treated subjects, and the efficacy across drugs makes the case that cholinesterase inhibition is a viable treatment strategy for AD [28]. The fifth and most recent antidementia drug to receive FDA approval is memantine. Memantine is a noncholinergic drug, acting instead at the NMDA class of glutamate receptor. In addition to showing clinical efficacy in human subjects with AD [29, 30], memantine has also been shown to improve cognition in murine models of cerebral amyloidosis. NMDA antagonism should therefore be considered among the possible mechanisms of action of treatments producing cognitive improvement in murine models of AD. Our *in vitro* experiments showed no evidence that CA acts by way of these established therapeutic targets since there was no effect on cholinesterase activity or glutamate neurotoxicity.

In addition to the established therapies just described, strategies aimed at preventing the accumulation of, or promoting the clearance of, A*β* are under study. Inhibitors of amyloid synthesis and immunization against A*β* have diminished brain pathology and yielded cognitive and behavioral improvements in murine models of AD. Although amyloid synthesis inhibitors such as Lilly semagacestat have yielded negative clinical results [31], antiamyloid immunotherapy (bapineuzumab) remains under development with some promising initial results [32]. “Antiamyloid” strategies, therefore, represent another potential mechanism of cognition-enhancing therapies in AD. Our results do not show an effect of CA on A*β* levels *per se* but suggest that CA may protect neurons from A*β*-induced neurotoxicity without actually changing brain levels of A*β*. Most current clinical trials are focused on suppression of A*β* levels, thus the neuroprotectant effect of CA described here represents a novel mechanism, potentially complementary to the drugs in development.

**Figure 6:**
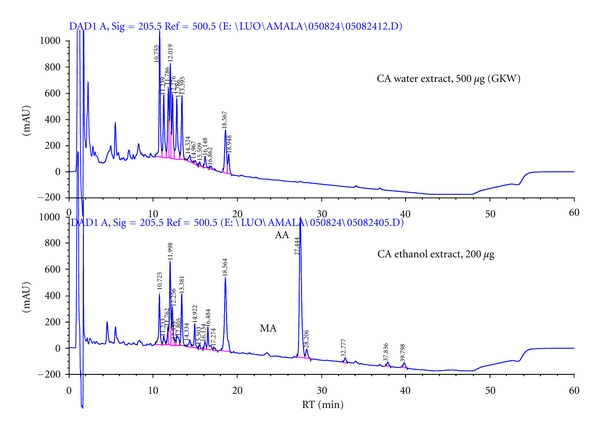
HPLC comparison of CA water and ethanol extracts made from the same batch of plant material. Asiatic acid (AA) and madecassic acid (MA) are detected in the ethanolic extract, but not in the water extract (GKW). The water extract GKW contains mostly very polar compounds as shown by their earlier elution than the triterpenes AA and MA. HPLC was conducted on an Aquasil 150 mm × 2.1 mm C18 column with acetonitrile:water gradient with 0.1% acetic acid. Acetonitrile concentration 10% at 0 min, 50% at 25 min, 90% at 40 min, then returning to starting conditions, detector wavelength 205 nm. AA and MA were identified by comparison of retention times to those of commercial standards.

CA may also be a source of a novel chemical class for the treatment of AD. HPLC analysis of GKW revealed a complex mixture of substances (Figure 6). This did not include asiatic acid or madecassic acid, well-known triterpene components of CA [33, 34], which were, however, extractable from the same plant material using ethanol (Figure 6). The absence of asiatic acid in GKW is notable since asiatic acid has been previously associated with neuroprotective and neurotropic effects [35–38]. However, an aqueous extract lacking asiatic acid produced robust behavioral effects in this study.  Table 1 lists the spectral characteristics of the major peaks found in GKW using LC-UV and LC-MS. UV spectra with maxima over 300 nm are indicative of a highly conjugated system, characteristic of flavonoids. CA is reported to be a rich source of quercetin [39]. Flavonoids isolated to date in CA include 3-glucosylkaempferol, 3-glucosylquercetin and diosmin [40, 41]. The molecular weights listed in Table 1 did not correspond to any of these 3 compounds, nor any other compounds isolated hitherto from CA (online chemical database, SciFinder). These findings imply the presence of potentially novel neuroactive ingredients in GKW which are yet to be fully characterized. 

Compared to the wealth of animal data described earlier, there have been fewer studies on cognitive effects of CA in humans. In one study, 30 mentally retarded children aged 7–18 years showed improvement in their general abilities after receiving 500 mg daily of dried CA herb for 3 months [42]. A more recent study [43] showed that an extract of CA (250–750 mg daily for 2 months) improved cognitive performance in healthy, elderly volunteers. In a placebo-controlled study, administration of CA herb (0.5 g/kg body weight) to healthy, middle-aged volunteers for 2 months resulted in improvements in several tests of cognitive function [44]. A study in elderly subjects with mild cognitive impairment found improvements in their cognitive test results, including the mini mental state examination, following administration of 500 mg dried CA twice a day for a 6-month period [45].

The traditional use of CA as an enhancer of cognitive function is therefore well supported by *in vitro*, *in vivo*, and small-scale human studies conducted so far. The ultimate goal of these studies is to develop evidence for the clinical use of CA, or compounds derived from CA, in the treatment or prevention of AD. The combination of data from *in vitro* and animal studies in the present work supports the impression that CA has the potential for clinical benefit in AD by way of a novel mechanism of action. Well-designed, controlled clinical trials of CA in AD and other forms of cognitive impairment are clearly warranted. The characterization of the active components of CA and elucidation of their mechanism of action would support these clinical studies.

## 4. Conclusions

A water extract of CA (GKW) attenuated A*β*-associated behavioral abnormalities in the Tg2576 mouse, a murine model of AD. *In vitro*, GKW protected SH-SY5Y cells and MC65 human neuroblastoma cells from toxicity induced by exogenously added and endogenously generated A*β*, respectively. GKW did not show anticholinesterase activity or protect neurons from oxidative damage and glutamate toxicity, mechanisms of current AD therapies. The combination of data from *in vitro* and animal studies in the present work supports the CA potential for conferring clinical benefit in AD, possibly by way of a novel mechanism of action. GKW does not contain asiatic acid, a known CA neuroprotective triterpene, but is rich in phenolic compounds. CA may therefore contain novel active compounds of relevance to the treatment of AD.

## Figures and Tables

**Table 1:** Molecular weight and UV data obtained for GKW components using LC-MS and LC-UV. Reversed phase gradient HPLC chromatography with UV and negative ion mass spectral detection was performed as described in Section 2.

Retention time	Most abundant ions (m/z); (Mol Wt−1)	UV *λ* max (nm)	Possible structure, based on flavonoid handbook [46]
12.33	399, 353	215, 325	Prenylated flavone
16.57	531	215, 265, 310	Malonyl, butyryl, or diacetyl flavone glycoside
21.05	477	205, 255, 355	Glycosyl or glucuronyl methylated flavones
22.64	561, 515	215, 325	Diglycosyl flavonoid or a catechin
25.21	601	215, 325	No matches found
33.49	577	220, 295, 320	Diglycosyl flavonoid or a proanthocyanidin
